# Development process and cognitive testing of CARATkids - Control of Allergic Rhinitis and Asthma Test for children

**DOI:** 10.1186/1471-2431-14-34

**Published:** 2014-02-06

**Authors:** Luís Miguel Borrego, João Almeida Fonseca, Ana Margarida Pereira, Vera Reimão Pinto, Daniela Linhares, Mário Morais-Almeida

**Affiliations:** 1 Immunoallergy Department, Hospital CUF Descobertas, Lisbon, Portugal; 2 CEDOC, Immunology Department, Faculty of Medical Sciences - New University of Lisbon, Lisbon, Portuga; 3 Allergy Department, Centro Hospitalar S. João E.P.E, Porto, Portugal; 4 Department of Health Information and Decision Sciences, Faculty of Medicine - University of Porto, Rua Dr. Plácido da Costa, 4200-450 Porto, Portugal; 5 Allergy Unit - Hospital and Instituto CUF, Porto, Portugal; 6 CINTESIS – Center for research in health technologies and information systems, Porto, Portugal; 7 Psychology Department, Hospital de Dona Estefânia, Lisbon, Portugal

**Keywords:** Asthma, CARATkids, Cognitive testing, Control, Pediatrics, Questionnaire, Rhinitis

## Abstract

**Background:**

Allergic rhinitis and asthma (ARA) are chronic inflammatory diseases of the airways that often coexist in children. The only tool to assess the ARA control, the Control of Allergic Rhinitis and Asthma Test (CARAT) is to be used by adults. We aimed to develop the Pediatric version of Control of Allergic Rhinitis and Asthma Test (CARATkids) and to test its comprehensibility in children with 4 to 12 years of age.

**Methods:**

The questionnaire development included a literature review of pediatric questionnaires on asthma and/or rhinitis control and two consensus meetings of a multidisciplinary group. Cognitive testing was carried out in a cross-sectional qualitative study using cognitive interviews.

**Results:**

Four questionnaires to assess asthma and none to assess rhinitis control in children were identified. The multidisciplinary group produced a questionnaire version for children with 17 questions with illustrations and dichotomous (yes/no) response format. The version for caregivers had 4-points and dichotomous scales. Twenty-nine children, 4 to 12 years old, and their caregivers were interviewed. Only children over 6 years old could adequately answer the questionnaire. A few words/expressions were not fully understood by children of 6 to 8 years old. The drawings illustrating the questions were considered helpful by children and caregivers. Caregivers considered the questionnaire complete and clear and preferred dichotomous over the 4-points scales. The proportion of agreement between children and their caregivers was 61%. The words/expressions that were difficult to understand were amended.

**Conclusion:**

CARATkids, the first questionnaire to assess a child’s asthma and rhinitis control was developed and its content validity was assured. Cognitive testing showed that CARATKids is well-understood by children 6 to 12 years old. The questionnaire’s measurement properties can now be assessed in a validation study.

## Background

Allergic rhinitis and asthma (ARA) are chronic inflammatory diseases of the airways that often coexist. The concept of “one airway one disease” was highlighted in the Allergic Rhinitis and its Impact on Asthma (ARIA) guidelines and the importance of an appropriate strategy combining safe and effective management of both diseases, targeting optimal control, in adults and children, was emphasized [1].

Questionnaires can be used as objective tools to evaluate disease control. For adults, there are several questionnaires that were developed to assess asthma control [2-4]. In what concerns allergic rhinitis the concept of control is still under definition [5]; nevertheless, some questionnaires have been proposed (Rhinitis Control Assessment Test (RCAT) [6] and Allergic Rhinitis Control Test [7]). For children, although the Practical Allergy (PRACTALL) consensus report [8] emphasized the use of these tools to monitor asthma control, few questionnaires are available [9-12]. And none to assess rhinitis control.

Based on ARIA recommendations, a single questionnaire should evaluate the control of asthma and allergic rhinitis [1,13]. To account for this need, the Control of Allergic Rhinitis and Asthma Test (CARAT) was developed and validated [14-16] and it has been proposed as the first tool implementing ARIA guidelines in clinical practice [13]. However, it was only validated for adult patients [17] and such combined tool was still missing for children. To address this problem we aimed to develop the CARATkids - Control of Allergic Rhinitis and Asthma Test for children – a questionnaire to concurrently assess control of allergic rhinitis and asthma in children under 12 years old, with a medical diagnosis of ARA. This article reports the process of development of the CARATkids and the results of the cognitive testing that lead to the preliminary version of the questionnaire.

## Methods

### Questionnaire development

**Figure 1:**
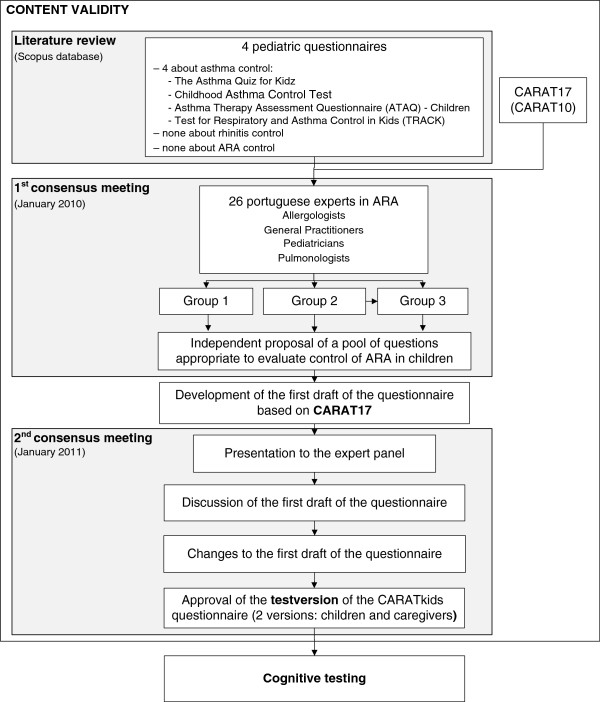
**Process of development of the CARATkids questionnaire.** The CARATkids questionnaire was developed in sequential steps, including literature review, 2 consensus meetings and a cognitive testing, to assure its content validity.

The development of the test version of CARATkids was performed in three steps (Figure 1), including a literature review and two consensus meetings.

### Literature review

A bibliographic search was conducted in MEDLINE using as search terms: child/children, asthma, rhinitis, questionnaire and/or control. Articles addressing questionnaire development and/or validation were selected and retrieved. References of the selected articles were searched for additional data sources. The data extracted included: age group and disease(s) of the target population, item generation methods, intended respondent, proportion of agreement between child and caregiver when applicable, number of questions, time frame, answering options (type of scales used), the presence of drawings and items assessed.

### First consensus meeting

A working group reviewed the existing data and proposed a pool of questions on control of ARA in children. This working group of 26 Portuguese experts in ARA, including Pediatricians, General Practitioners, Pulmonologists and Allergologists, participated in a consensus meeting, held in January 2010, where data on existing pediatric questionnaires and the initial (CARAT17 [14]) and reduced versions of CARAT (CARAT10 [15,16]) for adults were discussed. In this meeting, 3 groups independently analyzed the existing questionnaires and proposed questions to be included in CARATkids. After pooling the proposed questions, it was unanimously decided to use CARAT17 as the basis for the first draft version of CARATkids as it covered all the questions proposed (Figure 1).

The expert panel considered important that, at least during the development of the questionnaire, both child’s and their caregivers’ input were gathered using a similar set of questions. This would imply two versions of the questionnaire: one to be answered by the child and another by the caregiver.

### Second consensus meeting

In January 2011 the first draft of the questionnaire was presented to the expert panel. The draft was discussed, suggestions were presented, changes were made and a test version of the questionnaire approved (Figure 1).

This development process was designed to assure the content validity of the CARATkids questionnaire, according to the recently published COSMIN (COnsensus-based Standards for the selection of health Measurement Instruments) checklist [18].

### Cognitive testing

**Figure 2:**
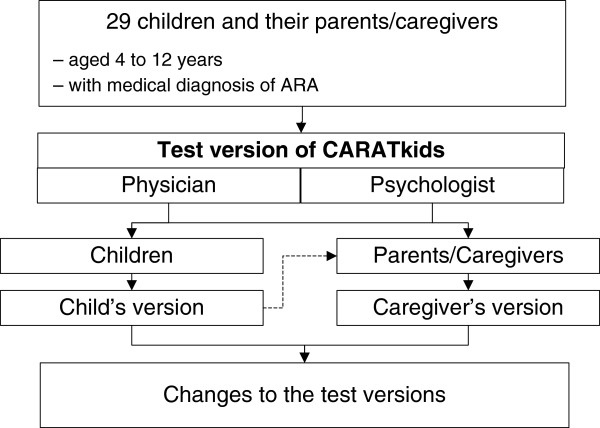
**Cognitive testing of the CARATkids questionnaire.** Cognitive interviews were made independently by a psychologist and a physician to each child and to one of his/her caregiver.

The cognitive testing was a cross-sectional, observational, qualitative study with face-to-face interview of a convenience sample of 29 children and their parents/caregivers. Children with 4 to 12 years old with medical diagnosis of allergic rhinitis and asthma (Figure 2) were enrolled between July and December 2011, during regular appointments at the Allergy outpatient clinic of the Pediatric Hospital Dona Estefânia, in Lisbon, Portugal; children and caregivers were eligible if they were native Portuguese speakers. The interviews were performed independently by a psychologist and a physician. Children and their caregivers were interviewed separately. The participants were asked to read each question and response aloud; when the children couldn’t read by him/herself, the questions were read aloud by the interviewers. The answers were then challenged to make sure that the questions had been correctly understood. The child’s version was also discussed with the caregivers.

This study was conducted according to the principles expressed in the Declaration of Helsinki. Verbal informed consent was obtained from the caretakers of all children participating in survey. These procedures were approved by the institutional Ethical Review Board of the Hospital CUF Descobertas, Lisbon, Portugal.

## Results

### Questionnaire development

Four questionnaires on asthma control were retrieved (The Asthma Quiz for Kidz [9], Childhood Asthma Control Test (C-ACT), Asthma Therapy Assessment Questionnaire (ATAQ) for Children and Adolescents [11], Test for Respiratory and Asthma Control in Kids (TRACK) [12]). No questionnaires on control of rhinitis or concurrent assessment of ARA for children were found.

Two versions of the CARATkids questionnaire were developed (child’s and caregiver’s) with slight differences in wording and response options.

### Child’s version

A 17-item questionnaire with dichotomous (Yes/No) answer format accompanied by an illustrative colored drawing was prepared. The dichotomous answer, similar to “The Asthma Quiz for Kidz” and ATAQ for children, was considered preferable for a child’s questionnaire as it is easy to understand and answer.

In the child’s version, the expression of a specific time-frame was included only in four questions. The expert panel considered the previous 2 weeks as the most adequate time-frame to be answered by children. In relation to CARAT17 for adults, questions’ and headings’ vocabulary was adapted and/or simplified to be more appropriate and easily understood by children (e.g.: “school” instead of work).

Regarding the colored drawings for each question, it was consensual to have only one version for both boys and girls; the image of a boy was selected. The drawings were discussed by the expert panel and improved until approval.

### Caregiver’s version

In the questionnaire for caregivers all questions had a time-frame of 2 weeks. The caregiver version of the questionnaire kept the format of CARAT17, namely a 4-points Likert scale in questions 1 to 15 and a dichotomous scale in questions 16 and 17. To make clear that this questionnaire related to the child, all the questions in the caregiver’s version included the expression “(…) your child (…)”.

### Cognitive testing

The characteristics of the children included in the cognitive testing are summarized in Table 1.

**Table 1:** **Characteristics of the children included in the cognitive testing** (**n** = **29**)

	**n**	**(%)**
**Gender**		
Female	11	(38)
**Age**, *years*, median (P25-P75)	8(6–10)	
4–5	3	(10)
6–9	15	(52)
10–12	11	(38)
**Ability to read**		
Without help	18	(62)
With help	11	(38)

Children with 4 to 5 years old were not able to read by themselves and could not fully understand the questionnaire. Children with 6 years or older were able to understand the questions; however, a few words, such as “rhinitis” or “symptom” were not known by children with 6 to 8 years. Moreover, the words/expressions “dyspnea” and “eye weep” were not understood by several children, irrespective of age, and were removed. A few words were replaced by synonyms that were more easily understood by children and the sentence “respiratory/allergic diseases” was abridged to “allergies”. All children older than 9 years considered the questionnaire very easy and clear. The children enjoyed the drawings and found them clear and illustrative of the concepts. None of the children or caregivers had questions or concerns about the drawings.

Caregivers agreed that the questionnaire was complete and no suggestion for additional questions were proposed. They considered that the 2 weeks period time was adequate. When considering the child’s version, caregivers reported it to be clear and adequate; they appreciated the dichotomic scale, considering it to be more appropriate for their children, compared to the 4-point Likert scale of the caregiver’s questionnaire. Most of the caregivers were in favor of the inclusion of time frame in the questions of the child’s version. Similarly, some children opposed to the absence of a time frame in the child’s version and older children specifically asked for how long should they think about; this fact may have caused some disagreement between the caregivers’ and child’s answers. Nevertheless, four (out of 6) of the 6 years old children didn’t understand the time-frame of 2 weeks.

The proportion of agreement between children and their caregivers was 61%; none of the child/caregiver pairs agreed in all the answers. The symptoms of nasal obstruction and throat itching were less reported by caregivers than by their children.

**Figure 3:**
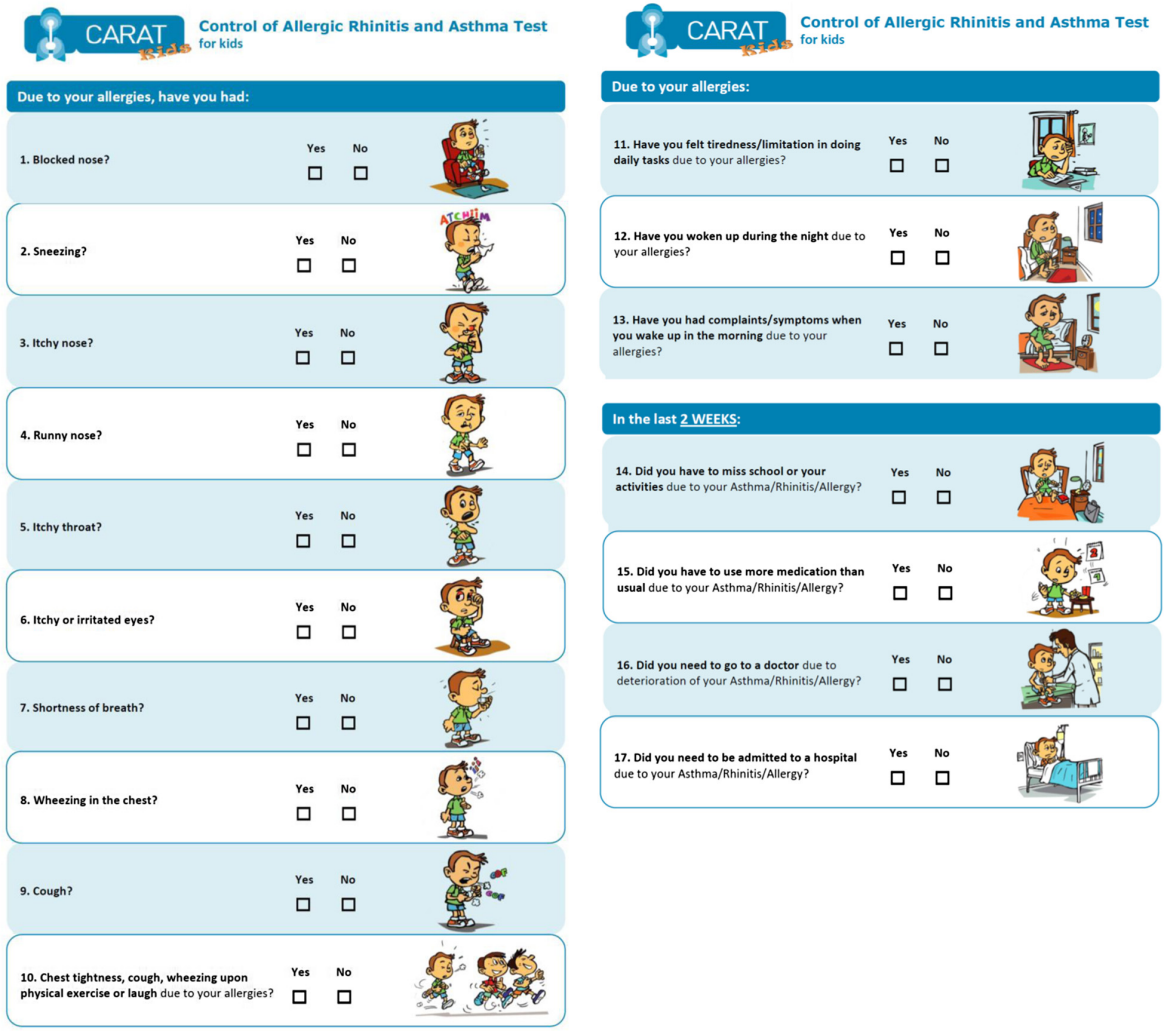
**Test version of CARATkids – child**’**s version.**

The test version for children, revised after the cognitive testing, is presented in Figure 3.

## Discussion

The process of development of the CARATkids questionnaire included literature review, two consensus meetings with a multidisciplinary expert panel and face-to-face interviews with children and their caregivers for cognitive testing. The cognitive testing showed that the test version of CARATkids was easily understood and answered by both caregivers and children with 6 years or older. Younger children (4 to 5 years) were not able to fully understand the questionnaire.

Most of the existing questionnaires evaluating asthma control in children were developed based on clinical guidelines (Table 2) and none assessed asthma and allergic rhinitis concurrently. Within available questionnaires, CARAT17 was the only one assessing the control of both asthma and allergic rhinitis and was based on a comprehensive item generation process [14] making it suitable to be the basis for development of CARATkids.

**Table 2:** **Characteristics of CARATkids** (**test version**) **and comparison with other questionnaires analyzed during the consensus meetings**

	**CARATkids (test version)**	**ATAQ-Children**	**C-ACT**	**The asthma quiz for kidz**	**TRACK**	**CARAT17**
Target population						
*Age group*	4-12 years	5-17 years	4-11 years	1-17 years	<5 years	Adults
*Disease* (*s*)	ARA	Asthma	Asthma	Asthma	Asthma	ARA
**Basis for questionnaire development**	CARAT17 (Literature review)	ATAQ for adults	NAEPP guidelines [19] GINA guidelines (Literature review)	Clinical criteria of the Canadian Asthma Consensus Statement [20]	Content generated from 2 focus groups^‡^ NAEPP guidelines [19]	Literature review with item generation
**Questionnaire**’**s structure**						
*Questions*, *n*	17	20^¥^	7 (4 + 3)*	6	5	17
*Input* (*versions*, *n*)	Child and caregiver (2)	Caregiver (1)	Child and caregiver (1*)	Child and caregiver (1)†	Caregiver (1)	NA
*Agreement* (*child*/*caregiver*)	61%	NA	80%	85%	NA	NA
*Time frame*	Questions 1-14: none Questions 15-17: 2 W	Recent symptoms: 4 W Chronic symptoms: 12 M	Child’s questions: present Caregiver’s questions: 4 W	Questions 1-4: last week Questions 5-6: preceding 30 D	Questions 1-3: 4 W Question 4: 3 M Question 5: 12 M	4 W
*Scale* (*s*)	Dichotomous	Dichotomous	4-point Likert	Dichotomous	5-point Likert	Questions 1–13, 15: 4-point Likert Questions 14, 16, 17: dichotomous
*Drawings*	Yes (questions)	No	Yes (answers)	Yes (questions)	No	NA
*Items*/*symptoms*	- Nasal obstruction	- Control:	- Child:	- Cough, wheeze or hard time breathing	- Wheezing, coughing or shortness of breath	- Nasal obstruction
	- Sneezes	-- Wheeze with exercise	-- Asthma today			- Sneezes
						- Nasal itching
	-Nasal itching	-Wheeze not exercising	--Asthma with exercise	- Wake up at night		- Nose dripping
					- Interference with exercise or usual activities	- Throat symptoms
	- Nose dripping					- Eye symptoms
	- Throat symptoms	-- School absenteeism	-- Cough	- Use of the blue puffer/pump		
					- Wake up at night	- Shortness of breath
	- Eye symptoms		-- Wake up during the night		- use of rescue medication	
	- Shortness of breath	-- Daily activity loss		- Cough, wheeze or hard time breathing with exercise (less exercise than previously)		- Wheezing
						- Chest tightness with efforts
	- Wheezing				- Use of oral corticosteroids	- Cough
						- Tiredness in daily activities
		-- Parent assessment of control	- Caregiver:			
						- Wake up during the night
	- Chest tightness with efforts		-- Daytime symptoms			
		-- Use of quick				- Work/School absenteeism
	- Cough					
			-- Wheeze during the day			
						- Increase the use of medication
	- Tiredness in daily					
						- Go to a doctor
						- Be hospitalized
	Activities					
	- Wake up during the night	Reliever	-- Wake up during the night	- School absenteeism		
		-- Dissatisfaction with treatment				
	- Work/School absenteeism			- Unscheduled medical appointment		
	- Increase the use of medication					
	- Go to a doctor					
	- Be hospitalized					
		-- Use of controller medication				
		- Attitude/behavior				
		- Self-efficacy				
		- Patient-provider communication				

*ATAQ* – Asthma Therapy Assessment Questionnaire (*ATAQ*) for Children and Adolescents; CARAT17 – Control of Allergic Rhinitis and Asthma Test (17-item version); *C*-*ACT* – Childhood – Asthma Control Test; *ACT* – Asthma Control Test; *TRACK* – Test for Respiratory and Asthma Control in Kids; CARATkids – Pediatric Control of Allergic Rhinitis and Asthma Test; *ARA* – Allergic Rhinitis and Asthma; *NAEPP* – National Asthma Education and Prevention Program; *GINA* – Global Initiative for Asthma.

*W* - week; *M* – month; *D* – day; *NA* – Not applicable.

¥ 7 on asthma control; * includes different questions for child, 4 questions, and caregiver, 3 questions; † when the child is 8 years or younger the questionnaire is completed by the caregiver with the child’s input; ‡ including physicians and primary caregivers of children with recurring respiratory problems or asthma.

Moreover, the expert panel and the caregivers (in the cognitive testing) had no suggestion for other questions to be included or existing questions to exclude: this supports the relevance of the included items.

The importance of incorporating the child’s perspective when evaluating asthma control has been highlighted in several studies [9,10]; however, in younger children (especially when they are unable to read by themselves or don’t know how to read properly), the parent/caregiver’s input may be essential [12]. In fact, we found that children younger than 6 were unable to adequately understand the questionnaire, suggesting that, in this age group, ARA control may be best assessed by a tool to be answered by the caregiver alone or with the child’s help. In older children, the independent input of children and caregivers ensures a broader perspective of diseases’ control and overcomes the limitations associated with relying in one isolated report, either from the child or the parent. Supporting this decision, the cognitive testing of CARATkids showed 39% disagreement between the symptoms reported by the child and caregiver; this was already reported by other studies [10,21]. The inclusion of nasal and throat symptoms (e.g.: nasal obstruction and throat itching) may have heightened this disagreement. These symptoms are frequently long-lasting but mild enough not to interfere majorly with the children’s daily activities; therefore, caregivers often don’t notice nasal or throat symptoms or regard them as “normal” or as part of a persistent cold and not as a sign of an allergic disorder. Nevertheless, the agreement between children and caregivers answers should be reassessed in future studies with the final version of the questionnaire.

The time frame used for assessment of control in existing pediatric questionnaires is highly variable and depends on the input (s) included (child vs. caregiver vs. child and caregiver). However it seems consensual that in child’s questions it is more appropriate and reliable to evaluate short time periods. Childhood-ACT [10], for example, uses “today” in questions to be answered for children because in a round of interviews with children aged 4–6 years, they presented difficulties recalling beyond 1 day. In the existing questionnaires, none of the questions to be answered by children evaluates more than 4 weeks (Table 2). In the development of the CARATkids questionnaire it was decided to eliminate the time frame from most of the questions based on the knowledge that children, especially the younger/pre-school, may be unable to remember or even understand the concept of one week or one month. Only those questions regarding the use of rescue medication, unscheduled medical visit and hospital admission (more “relevant” events) reported specifically to the last 2 weeks. This option was very much discussed during consensus meetings and by some children and caregivers in the cognitive interviews and should be further studied.

The CARATkids questionnaire was designed with a dichotomous-scale answer format, similar to The Asthma Quiz for Kidz [9] and ATAQ for children [11]. This answer format was considered adequate by children and parents.

The inclusion of drawings illustrating each question was also appreciated by the participants. This kind of visual support was already used in The Asthma Quiz for Kidz [9]; the C-ATC [10] also includes drawings, but only to illustrate the answer options. It was consensually decided to use the image of a boy and neither children nor caregivers participating in the cognitive testing of CARATkids reported questions related to the use of a boy’s image; previous research [10] has shown that the boy’s face is preferred by the majority of children (vs. generic or girl’s face).

The cognitive test assured that the questionnaire was adequately understood by both children and caregivers and allowed to improve words and expressions that were not sufficiently clear. This was a fundamental step in the development of this questionnaire, providing in-depth data, that was a major contribute to build a clear and appealing questionnaire. Moreover, it allowed the assessment of its feasibility in the target age groups. The comments of the caregivers supported the idea that CARAT is an useful and adequate questionnaire to assess asthma and rhinitis control. This process may seem limited by the small number of participating children, especially in the group with 4 to 5 years old. However, as a qualitative methodology, cognitive interviews should be stopped when no more new information is being obtained. For children with 4 to 5 years old with a few interviews was clear that the type of questionnaire we were developing could not be applied. Therefore we stop to interview children from that age group.

As far as we know, CARATkids is the first questionnaire aiming to assess control of asthma and allergic rhinitis concurrently in children. It is being developed following the COSMIN check-list [18] that evaluates the methodological quality of studies on measurement properties. Although this is not specific for childhood questionnaires, we believe that the COSMIN initiative [18] can help to improve the development and selection of health measurement tools. Additional knowledge, specifically regarding the development of pediatric questionnaires, should be sought.

This article describes the aspects assuring the content validity [18] of the CARATkids questionnaire. Additional studies are needed to evaluate its measurement properties. A clinical prospective validation study is underway.

## Conclusion

CARATkids, the first questionnaire to assess a child’s asthma and rhinitis control was developed and its content validity was assured. Cognitive testing was important to guarantee that CARATKids is well-understood by children 6 to 12 years old. The questionnaire’s measurement properties can now be assessed in a prospective validation study.

## Abbreviations

ARA: Allergic rhinitis and asthma; ARIA: Allergic rhinitis and its impact on asthma; ATAQ: Asthma therapy assessment questionnaire; C-ACT: Childhood asthma control Test; CARAT: Control of allergic rhinitis and asthma test; CARATkids: Pediatric version of the control of allergic rhinitis and asthma Test; COSMIN: COnsensus-based standards for the selection of health measurement instruments; GINA: Global initiative for asthma; NAEPP: National asthma education and prevention program; PRACTALL: Practical allergy; RCAT: Rhinitis control assessment Test; TRACK: Test for respiratory and asthma control in kids.

## Competing interests

The authors declare that they have no competing interests.

## Authors’ contributions

LMB participated in the study conception, data collection and interpretation and wrote the manuscript draft, JAF participated in the study conception, data interpretation and in the writing of the manuscript, AMP and DL participated in data interpretation and in the writing of the manuscript draft, VRP participated in the data collection, MMA participated in the study conception and provided critical review during the project. All authors have reviewed and approved the final manuscript.

## Pre-publication history

The pre-publication history for this paper can be accessed here:

http://www.biomedcentral.com/1471-2431/14/34/prepub
